# Cellular senescence and kidney aging

**DOI:** 10.1042/CS20230140

**Published:** 2023-12-21

**Authors:** Nikolai Rex, Anette Melk, Roland Schmitt

**Affiliations:** 1 Department of Nephrology and Hypertension, Medical School Hannover, Germany; 2 Department of Pediatric Kidney, Liver and Metabolic Diseases, Medical School Hannover, Germany; 3 Department of Nephrology and Hypertension, University Hospital Schleswig-Holstein, Kiel, Germany

**Keywords:** Aging kidney, Cellular Senescence, chronic kidney disease, senolysis, senolytic therapy

## Abstract

Life expectancy is increasing worldwide, and by 2050 the proportion of the world’s population over 65 years of age is estimated to surpass 1.5 billion. Kidney aging is associated with molecular and physiological changes that cause a loss of renal function and of regenerative potential. As the aging population grows, it is crucial to understand the mechanisms underlying these changes, as they increase the susceptibility to developing acute kidney injury (AKI) and chronic kidney disease (CKD). Various cellular processes and molecular pathways take part in the complex process of kidney aging. In this review, we will focus on the phenomenon of cellular senescence as one of the involved mechanisms at the crossroad of kidney aging, age-related disease, and CKD. We will highlight experimental and clinical findings about the role of cellular senescence in kidney aging and CKD. In addition, we will review challenges in senescence research and emerging therapeutic aspects. We will highlight the great potential of senolytic strategies for the elimination of harmful senescent cells to promote healthy kidney aging and to avoid age-related disease and CKD. This review aims to give insight into recent discoveries and future developments, providing a comprehensive overview of current knowledge on cellular senescence and anti-senescent therapies in the kidney field.

## Introduction

Today, life expectancy in industrialized nations has reached an unprecedented peak, yet it continues to rise [1]. This trend is expected to persist on a global scale throughout this century. The upturn in longevity will result in a substantial increase in the proportion of the population aged 65 and above, potentially reaching over 1.5 billion by 2050 [2]. Aging is a complex biological process characterized by a progressive decline in physiological function and an increased susceptibility to disease. This process is accompanied by functional and structural changes in the body. The underlying mechanisms are the subject of intense research [3].

The kidney, a vital organ for waste excretion and blood filtration, naturally changes during life. With advanced age, the kidney undergoes structural changes such as glomerulosclerosis, tubular atrophy, arteriosclerosis, and interstitial fibrosis [4]. These changes are associated with decreased kidney function, most easily noticed by a reduced glomerular filtration rate (GFR), and increased susceptibility to acute kidney injury (AKI) and chronic kidney disease (CKD) [5].

Cellular senescence is defined as a state of stable arrest of the cell cycle and was identified as a key factor in aging and age-related diseases [6]. Originally observed by Hayflick and Moorhead in cell cultures [7] and eventually also found *in vivo*, senescence is a response to various stressors that limits the division of aged or damaged cells. Senescent cells undergo significant changes, including metabolic reprogramming and modulation of autophagy, and secrete a heterogenic variety of bioactive molecules known as senescence-associated secretory phenotype (SASP) that affect neighboring cells and may contribute to chronic inflammation [8,9].

Senescence was initially regarded as a cancer suppressive mechanism but has now also been recognized as an important promoter of aging and age-related diseases [10,11]. Senescent cells accumulate in tissues over time and can impair tissue function by loss of their normal cell type-specific function, loss of mitotic capacity, and SASP [12]. There is strong experimental evidence that the accumulation of senescent cells contributes to the functional decline observed during aging. With the improved understanding of mechanisms of cellular senescence and the discovery that the removal of senescent cells in animal experiments (senolysis) prolongs health parameters and lifespan, the question of intervention options at the junction of senescence and age-related diseases arose [13].

This review focuses on the current understanding of the connection between aging and cellular senescence in the physiologically aged and in the diseased kidney. It aims to give insight into recent groundbreaking discoveries and identifies future challenges that we need to address before the successful translation of anti-senescent therapies in the clinical context of kidney diseases.

## Structural and functional changes of the aging kidney

**Figure 1:**
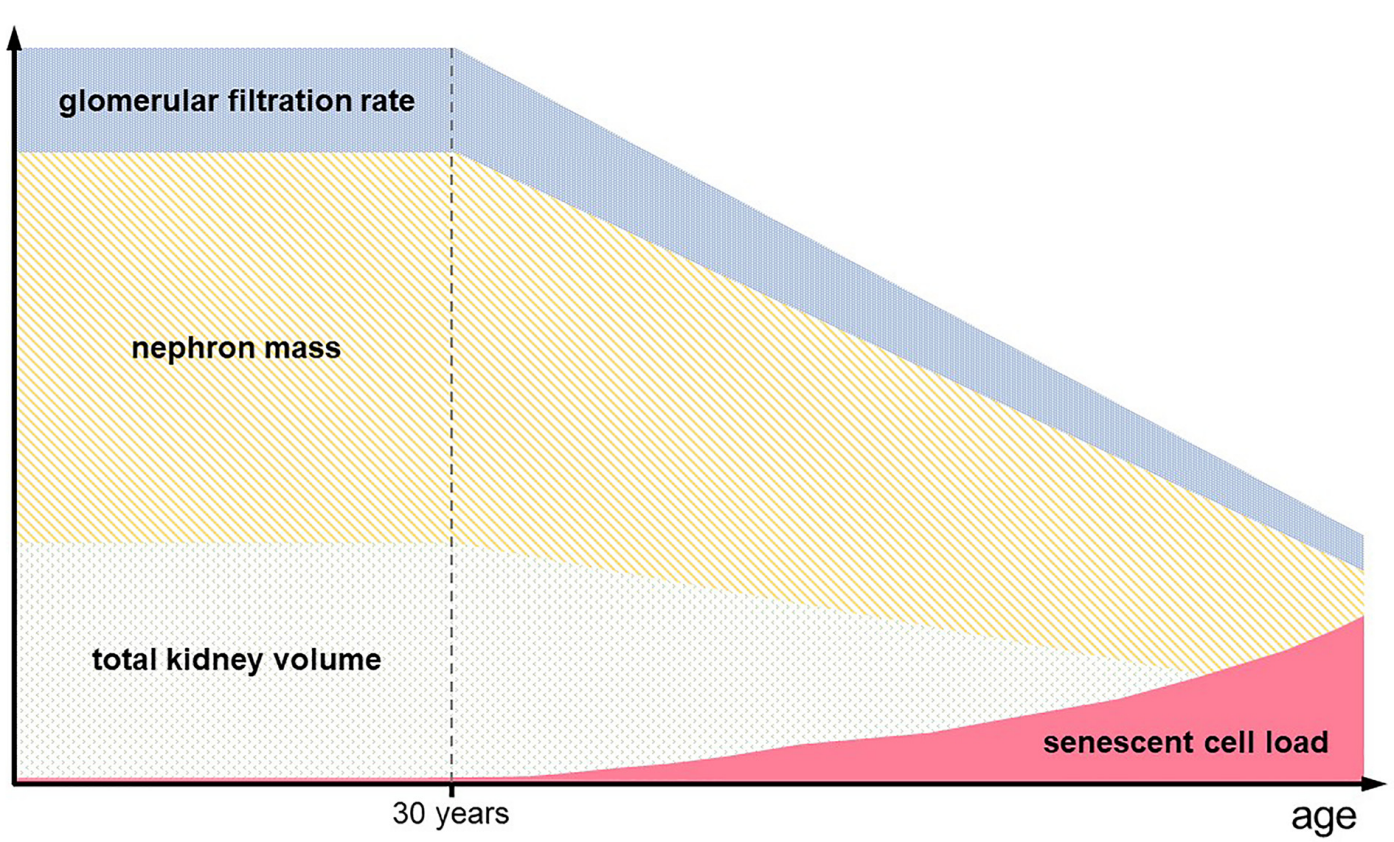
Age related changes in kidney function and structure With natural aging, there is a loss of nephron mass (yellow) and total kidney volume (green) starting around the age of 30 years. This is paralleled by a steady decrease in glomerular filtration rate with a loss of approximately 1 ml/min/year (blue). The load of senescent cells within the kidney increases with age (red).

As individuals age, substantial functional and structural alterations occur within the kidneys. Despite the abundant endowment of nephrons we are born with, their ongoing loss throughout adult life leads to diminished regenerative potential, which amplifies vulnerability to further damage, even among healthy individuals. In the third decade of life, the GFR starts to decline at a rate of roughly 1 ml/min/1.73 m^2^ per year [14,15] (Figure 1). In parallel, the kidney’s parenchymal volume decreases at a measurable pace, with differing trajectories for the renal cortex and medulla. An accelerated decrease in cortical volume becomes evident from 50 years of age onwards. The volume of the medulla, on the other hand, displays a sex-specific pattern after the age of 50, with a further increase in men but a starting decline in women [16]. Biopsy studies of healthy living kidney donors have demonstrated a 48% loss of nephrons between the youngest (18–29 years) and oldest (70–75 years) donor groups, mirroring the decline in GFR across an adult’s lifetime. This was accompanied by a 15% increase in globally sclerotic glomeruli and a 16% decrease in cortical mass [17].

At present, no clear boundary exists between age-related kidney changes and the diagnosis of CKD. The most accepted histological feature defining CKD is a combination of glomerulosclerosis, tubular atrophy, tubulointerstitial fibrosis (TIF), and arteriosclerosis. This combination is commonly described as nephrosclerosis, and in the case of the aging kidney, also known as senile nephrosclerosis [18]. In the clinical context, ‘benign’ nephrosclerosis is often distinguished from ‘malignant’ nephrosclerosis, with the latter referring to the typical damage pattern of accelerated hypertension. Surprisingly benign or senile nephrosclerosis was found in 73% of healthy individuals aged 70–77, without an associated increase in End Stage Renal Disease (ESRD) risk or mortality. These findings prompt further questions regarding the distinction between healthy physiological and pathological kidney aging [4,19].

Through biopsy studies of living kidney donors, several histologic markers indicative of pathological kidney aging have been identified. An increase in single nephron GFR, associated with CKD risk factors regardless of age, stands out as a notable marker [20]. The presence of globally sclerotic glomeruli beyond the 95th percentile also indicates chronic pathological injury [21]. By assessing the prevalence of glomerulosclerosis in the renal cortex, it seems to be possible to discern whether the changes or damage are strictly age-related. Superficial glomerulosclerosis is most closely linked to age, while conditions like hypertension, proteinuria, interstitial fibrosis, and low GFR consistently show sclerosis patterns across the cortex [22]. An essential cell type in age-related glomerulosclerosis are podocytes, which are highly specialized cells covering the glomerular capillaries. Biopsy studies revealed a substantial age-related reduction in podocyte density associated with podocyte hypertrophy. In some cases, alongside the accumulation of proteinaceous material in Bowman’s space and increased cell stress markers, significant podocyte detachment and periglomerular fibrosis were observed [23].

### Aging is a risk factor for chronic kidney disease (CKD)

Aging is one of the principal risk factors for the onset of most chronic diseases, including CKD [24]. Despite advancements leading to longer life expectancy, there has been little progress in extending the health span of specific organ systems, a problem anticipated to remain [25]. CKD exemplifies the trend of an increase in age-related diseases. Age-associated, very common health conditions such as hypertension, diabetes, cardiovascular disease, and potential exposure to nephrotoxic medications cumulatively increase the risk for AKI and contribute to the transition from AKI to CKD [4,26–28]. The Kidney Disease Improving Global Outcomes (KDIGO) defines CKD, irrespective of its cause or underlying disease, as a chronic decrease in GFR to less than 60 ml/min per 1.73 m^2^, or the presence of kidney damage markers for at least three months. Aging stands out as the most powerful predictor for developing CKD [29,30]. Although CKD already contributes significantly to global morbidity and mortality, it poses an escalating challenge. Projections suggest that by 2040, CKD could rise from its 2016 rank of 16th to become the 5th leading cause of Years Life Lost (YLL) [31].

## Understanding cellular senescence

Before we discuss the role of cellular senescence in kidney aging and disease, we will provide an overview of the general principles of cellular senescence. Teleologically, senescence plays an important role as a cellular protection mechanism. It is triggered when injurious stress, organelle dysfunction, or DNA damage reach a critical threshold. Among a variety of stimuli, which can lead to the state of senescence, two main types of senescence induction can be distinguished: (i) replicative senescence, which is caused by telomere shortening, and (ii) damage-induced senescence, which can be triggered by factors such as oxidative stress, oncogene activation and cell culture stress in primary cells [10,32,33].

**Figure 2:**
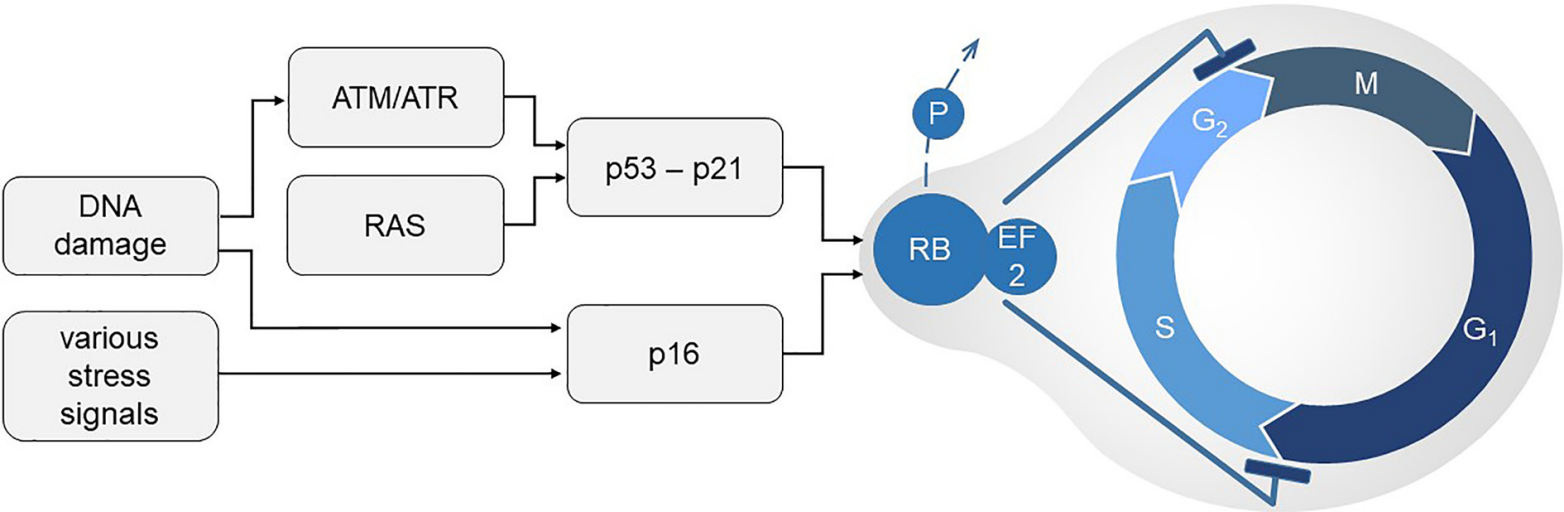
Pathways of cellular senescence DNA damage and a variety of stresses can activate the cellular senescence program. DNA damage activates Ataxia-telangiectasia-mutated (ATM) and ataxia telangiectasia and Rad3-related (ATR). ATM/ATR activate p53 and its transactivational target p21. Alternatively, activation of the RAS oncogene can also result in p53-p21 activation. Various stress signals can activate p16INK4a (p16), which similar to p21 is a cyclin-dependent kinase inhibitor. Both pathways converge on antagonizing the phosphorylation of tumor suppressor retinoblastoma (RB). Hypophosphorylated RB promotes cell cycle arrest by complexing EF2. Progression through the G1, S, G2, and M phases of cell division is halted at G1/S and G2/M.

Despite the different triggers for senescence, there are common molecular pathways involved, particularly the p53 and p16INK4a (subsequently abbreviated as p16) pathways and the upstream activation of Ras and Ataxia-telangiectasia-mutated (ATM) and ataxia telangiectasia and Rad3-related (ATR), which eventually converge on the blockade of retinoblastoma protein (RB) phosphorylation [34]. When RB is hypophosphorylated, it inactivates CDK–cyclin complexes and is tightly bound to the transcription factor E2F, which is needed for the cell to proceed with cell cycling (Figure 2). Prolonged RB-dependent cell cycle arrest is accompanied by specific intracellular changes, such as chromatin reorganization and the formation of senescence-associated heterochromatic foci (SAHF) [35]. The ATM/ATR pathway, activated in response to DNA damage, leads to the stabilization and activation of p53, which in turn activates p21, a cyclin-dependent kinase inhibitor that blocks RB phosphorylation [36]. The Ras signaling pathway, frequently activated by oncogenes, induces senescence through the activation of p53 and the production of reactive oxygen species [11]. The second major way to inhibit cyclin-dependent kinases, which normally phosphorylate RB, is the p16 pathway, which can be triggered by various stress signals [13,37] (Figure 2).

### General characteristics of senescent cells

Senescent cells are resistant to apoptosis due to the upregulation of prosurvival pathways, for example, members of the BCL-2 protein family, which contribute to their long-term persistence in tissues [38]. As discussed later in this review, the apoptosis resistance is one of the main targets for anti-senescence therapies (senolysis). Another characteristic feature of cellular senescence is the SASP. The SASP describes a secretion of proinflammatory cytokines, chemokines, growth factors, and proteases that affect surrounding cells and tissues [39]. While the SASP may physiologically stimulate the auto-clearance of senescent cells by the immune system, it may become detrimental in the context of age-related disease, contributing to the systemic microinflammatory state of aging and to chronic organ dysfunction. It is through the SASP that relatively small amounts of senescent cells might be capable of disturbing the function of entire organ systems. The endocrine character of the SASP might further explain the experimental finding that the transfer of senescent cells in the peritoneal cavity of young mice can cause remote organ dysfunction [40]. It is important to note that the SASP, just as other typical features of senescence, may strongly vary depending on the cell type [28,41,42]. This might be particularly important for the kidney, which consists of dozens of highly specialized cell types with a high degree of functional variations [43]. The SASP’s heterogeneity also appears to vary depending on the senescence inducer and the duration of senescence. Our understanding of these variations is still limited.

Among several molecular pathways controlling the SASP, p53, and p16 pathways [44] and the DNA damage response (DDR) are significantly involved, although details about their individual roles in the kidney are yet to be clarified [45]. The DDR activates ATM, leading to NFκB activation as a major SASP regulator with a strong association to kidney aging [46]. The NFκB pathway drives the expression of many SASP components, including IL-6 and IL-8 [47]. Single-cell transcriptomic findings identified failed proximal tubule repair cells after AKI as a pro-fibrotic, pro-inflammatory cell type with marked activation of NF-κB and expression of SASP genes [48].

Recently, the cGAS-STING (cyclic GMP-AMP synthase-stimulator of interferon genes) pathway was recognized as a contributor to SASP induction and regulation [49]. Although its direct involvement in renal senescence has not been shown yet, it is known that cytosolic chromatin fragments sensed by cGAS in senescent cells trigger a type-I interferon response, contributing to SASP and promoting paracrine senescence [49]. The mechanistic target of rapamycin (mTOR) pathway also influences SASP activation in senescent cells. Inhibition of mTOR signaling with rapamycin has been shown to suppress the secretion of many SASP factors [50]. However, the intricate interplay between these different SASP-inducing pathways and their relative contributions to aging and to different pathologies is presently still unclear.

### Pathways and checkpoints leading to cellular senescence

Regular cell cycling requires passage through various cell cycle checkpoints. When DNA damage or other irregularities necessitating repair occur, the cell cycle usually halts. The first important halt is the G1 arrest (transition from G1 to synthesis phase is stopped), which serves as a crucial checkpoint controlling entry into S phase, where DNA replication occurs [51] (Figure 2). Failure of the G1 checkpoint can lead to uncontrolled cell proliferation and also increased cancer risk [52]. Age-related renal cellular senescence is mainly associated with a cell cycle halt in the G1 phase via the above-mentioned pathways promoting a G0/G1 arrest and eventually a sustained cell cycle exit [53]. The G1 arrest is characteristic for most senescent cells and is typically accompanied by defined senescence-associated features, such as increased senescence-associated β-galactosidase (SA-β-GAL) activity. Pathways associated with cellular senescence and G1 arrest include p53, plasminogen activator inhibitor-1 (PAI-1), nuclear lamin B1 (LB1), ROS signaling, p15Ink4b, p21, p16, and the PI(3)K-PKB-GSK3β pathway. LB1 expression decreases during cellular senescence, and its silencing can trigger premature senescence via the p53 and RB signaling pathways [54–57].

The second prominent cell cycle halt, which became increasingly relevant in the kidney field, is the G2/M arrest (transition from G2 to M phase is stopped). The G2/M arrest plays a critical role in preserving genomic integrity by preventing cells from entering mitosis [58]. The kinases ATM and ATR are key regulators at this checkpoint, with DNA damage activating ATM and replication stress activating ATR [59]. Failure of the G2/M checkpoint can lead to genomic instability and cancer [60]. Although cellular senescence can occur in form of a G2/M arrest, it is important to note that even long-term G2/M arrest does not necessarily lead to cellular senescence [61], but instead, is often reversible and may transit into re-entry of cell cycle progression or into apoptosis [62]. In tubular epithelial cells, acute injury can induce a G2/M arrest (detectable by p-H3 antibodies), which is associated with the secretion of pro-fibrotic cytokines [63]. While it is currently unclear whether this AKI associated G2/M arrest is only senescence-like or corresponds to bona fide cellular senescence, recent findings show that the occurrence of G2/M arrested cells can be reduced by inhibition of HDAC9, a histone deacetylase that modifies chromatin organization and regulates Stat1 activation. This intervention rescued post-injurious tubular integrity and diminished fibrosis [64], highlighting that antagonizing the G2/M cell cycle arrest has therapeutic potential. Whether similar strategies would also be applicable to other forms of cell cycle arrest (e.g., G1 arrest) is presently unknown. Of note, the double knockout of HDAC1 and HDAC2 in podocytes had the opposite effect, causing a p21-mediated cell-cycle arrest in G1, resulting in senescence, SASP activation, and progressive glomerulopathy [65].

### Biomarkers used to detect senescent cells: an ongoing challenge

Although the understanding of the mechanisms of cellular senescence is the subject of intensive research, the detection of senescent cells is still a major challenge, especially *in vivo*. The main limitations consist in the lack of universal markers, the heterogeneity and complexity of senescent cells concerning their phenotypes and molecular profiles, the lack of standardized protocols leading to variability in results across different studies, the difficulty of detecting senescent cells in complex tissues due to a multitude of cell types and complex spatial distribution [66–68]. The most indisputable prerequisite but also least specific marker of cellular senescence is the lack of cell cycling/proliferation signs (e.g., Ki67), which should be absent in all states of senescence. Another strong marker is a critical shortness of telomeres, which can be measured by quantitative PCR or by fluorescence in situ hybridization (FISH) for spatial resolution [65]. A drawback of telomere length determination is the fact that it is mostly detecting replicative senescence and not stress-induced senescence, and that it cannot be used in standard rodent models, because telomere length inversely correlates with lifespan and mice and rats have 5- to 10-fold longer telomeres than humans [69]. Therefore, rodent telomeres do not reach a critical shortness during average lifetime.

SA-β-GAL activity at pH 6.0 is a widely used marker for classic G1/G0 exit cellular senescence. However, as it reflects increased lysosomal activity, it can give false-positive results due to high lysosomal activity under other conditions than senescence, including treatment with inhibitors of mitogen-activated protein kinases or CDK4/6 [70–72].

Among the most reliable markers of senescence is the expression of cell cycle inhibitors (cyclin-dependent kinase inhibitors), such as p16, p27, and p21, which can be monitored on the transcriptional or protein level. However, just like SA-β-GAL, these markers are not specific for senescent cells [66]. Compared with p21, p16 is generally believed to have a closer relationship to cellular senescence. However, there is clear evidence that the expression of p16 can also occur independent of senescence. A particularly strong expression has been shown in macrophages and mesenchymal cells [73,74], and a transient p16 expression can also be observed in parenchymal kidney cells, for example, in the context of ischemia/reperfusion injury [75] (unpublished own results). As CDKN2A (gene coding for p16) is transcribed in a comparatively low copy number, it is difficult to visualize gene expression by *in situ* hybridization, and the amount of transcript is usually too small to be captured by current single-cell sequencing methods [76,77]. Therefore new gene sets to identify senescent cells in transcriptomic data are being developed [76,78].

Similarly, histone proteins, such as phosphorylated γH2A.X, which are formed in the case of DNA damage, are present in the nuclei of senescent cells, but are not unique to cellular senescence, and are also found in all kinds of DNA stress and during regular cell proliferation [79,80].

Thus, it is crucial that the analysis of cellular senescence follows a standard combining the detection of different features: (i) proof of absence of proliferative features, (ii) expression of known genes associated with senescent cell cycle arrest (including negatively associated genes such as LB1), (iii) SASP candidate detection on the transcriptional or protein level, (iv) presence of DNA damage makers, (v) increased lysosomal activity (SA-β-GAL), and, in case of replicative senescence in humans, (vi) shortened telomere length [76,81].

**Figure 3:**
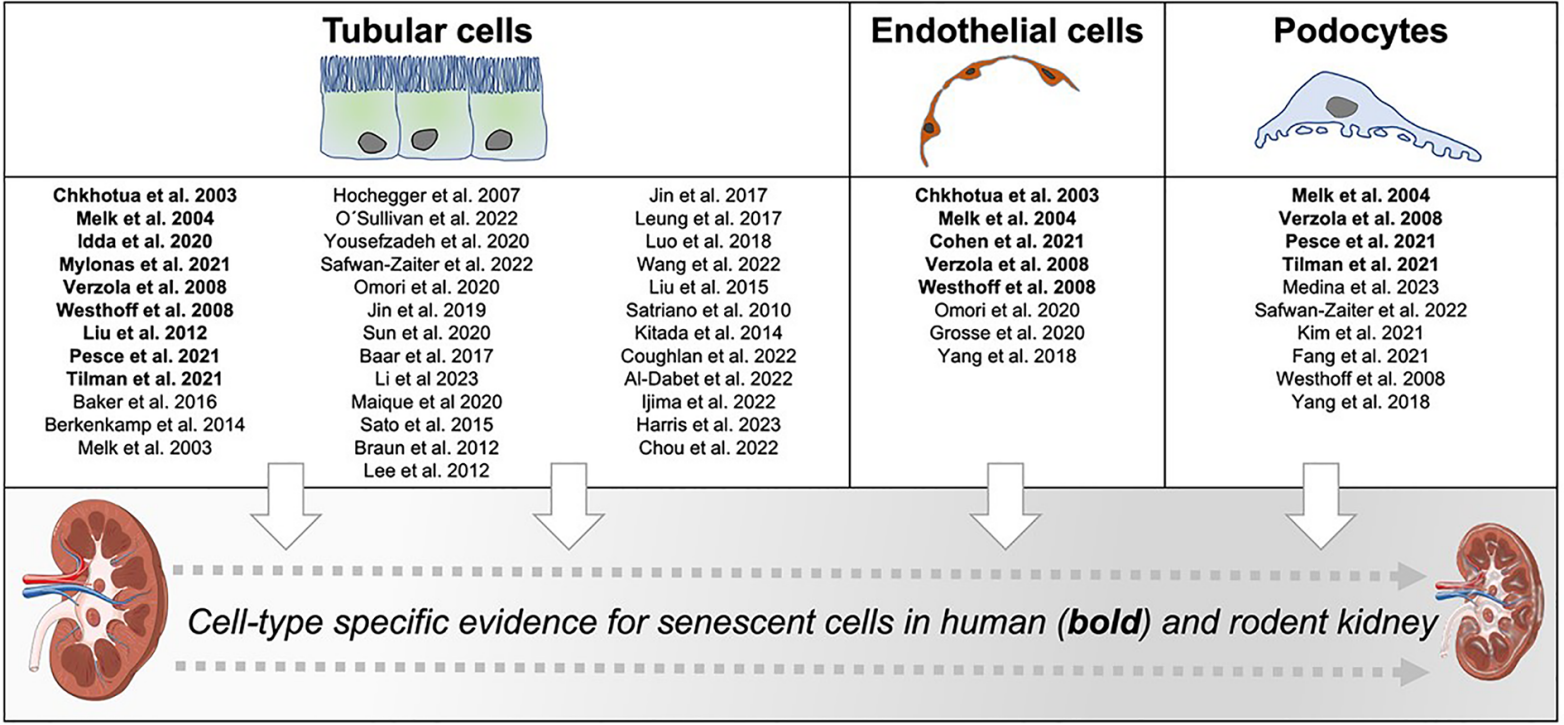
Major kidney cell types are affected by cellular senescence Evidence from the literature for senescence in indicated cell types is presented for tubular cells, endothelial cells and podocytes. References including human material are in bold.

Given the challenges of detecting senescent cells, it is not surprising that there is conflicting data about affected cell types in aging kidneys. Whereas many studies focused on tubular epithelial cells, the large majority of all cells in the kidney, there is accumulating evidence that podocytes and endothelial cells develop cellular senescence with crucial functional consequences [82,83] (Figure 3). Promising initiatives, such as the National Institutes of Health funded SenNet (Cellular Senescence Network), are expected to provide more comprehensive data on tissue mapping and characterization of cellular senescence in different organs and different species.

## Cellular senescence in the aging kidney

### Senescent cells in human kidney aging

To what extent do senescent cells contribute to the aging process of the kidney? A first approach to this question is the correlation between aging and the increasing number of senescent cells in the kidney. Due to the above-mentioned problems in detecting senescent cells, surprisingly little data are available on this issue. In human kidneys, Melk et al. showed for the first time in 2000 that telomere length is decreasing in an age-dependent fashion [84]. The age-correlation of telomere attrition was later confirmed by different groups in zero-hour biopsies of renal transplants, where shorter telomeres correlated with donor age and also predicted a reduced transplant outcome [85,86]. Another study explored cellular senescence in human kidneys, comparing the expression of p16 in kidney tissues of varying ages (21–80 years). The findings indicated that senescent cells progressively accumulate in the cortical tubules as age advances [87]. Simultaneous studies using p16 and SA-β-GAL demonstrated a similar age-related increase of senescent cells in the glomeruli, tubulointerstitium, and arteries of the human kidney [88,89]. An increased expression of p16 (or the corresponding gene CDKN2A) in older kidneys was corroborated in zero-hour biopsies of kidney transplants [85,86]. Idda et al. systematically tested the age-associated accumulation of senescent cells using human tissue arrays across a broad spectrum of ages. By immunohistochemistry, they demonstrated more p16 positive cells in older kidneys, but the general number of positive cells was very small and increased only moderately with age, reaching a total of 0.2% of cells at old age. Parallel findings were reported for p21 with a slightly higher increase of cells (up to 1% of all cells in old kidneys) [90].

### Senescent cells in rodent kidney aging

In aged mice and rats, there have been many studies showing increased kidney expression of p16, p21, SASP factors, as well as enhanced SA-β-GAL activity [33,88,91–93]. While murine p16 is difficult to visualize by immunohistochemistry, there are now several p16-based reporter mouse models, which can be used to follow p16 expression during aging and in disease models. However, the reporter strains do not all target the same p16-expressing subsets of cells due to different transgene designs, which can lead to discrepancies in the observed cell proportions and cell distributions [94–96]. Omori et al. found in a p16-CreERT2-tdTomato reporter mouse enrichment of p16 expression already in medium-aged mice (12 months) in kidney endothelial cells, renal proximal and distal tubule epithelia, while others observed expression only later and mainly in endothelial or in proximal tubular cells [97–99]. As a possible alternative for the p16 promoter, Sun et al. used the Glb1 gene locus for activation of a senescence reporter, which encodes lysosomal β-galactosidase (enzyme activity of SA-β-GAL staining). Glb1+/m‒Glb1-2A-mCherry reporter mice displayed reporter expression in kidney tubular cells with a positive age-correlation in middle-aged mice [99]. However, while the amount of expression of Glb1 correlates with cellular senescence, it is also constitutively expressed in non-senescent cells, making interpretation of these results difficult.

### Complexity of acute versus persistent senescent cell state

The impact of senescent kidney cells is likely to differ not only depending on the affected cell type but also on dynamic changes during the aging process and in the course of different disease states. While it is widely accepted that chronic persistence of senescent cells has negative effects, acute cellular senescence might be beneficial under some circumstances. For example, the cell cycle arrest of acutely injured tubular cells may help to avoid uncontrolled mitosis by providing more time for DNA repair. This concept is corroborated by several mouse studies, showing that cell cycle inhibition by administration of CDK4/6 inhibitors, p21 activation, or remote ischemic preconditioning is beneficial [100–102]. Supporting such heterogeneous effects, a recent AKI study in p16-reporter mice demonstrated an early induction of senescence (within 1–3 days) predominantly in proximal tubular cells. p16 expressing cells spontaneously vanished after two weeks, whereas other cells that occurred during a subsequent wave persisted and were associated with poor recovery and progression to CKD [103].

## Functional consequences of senescent cells in kidney aging, evidence from senolytic strategies

### Transgenic models of senolysis

Despite the above-mentioned shortcomings, p16-based transgenic strategies have enormously pushed the field, providing groundbreaking results on the functional role of cellular senescence in the determination of lifespan and organ aging. Baker et al. introduced p16-INK-ATTAC mice, a model in which the p16 promoter drives not only a reporter gene but also the expression of a suicide transgene, which can be activated by injection of a small molecule (AP20187). Using this strategy, the authors first showed in progeroid mice and later in naturally aged mice that the selective killing of p16-high cells suppresses age-related disease and prolongs median life span [13,104]. Ablation of senescent cells in aging p16-INK-ATTAC mice significantly reduced glomerulosclerosis, indicating a contribution of p16-high cells to this common hallmark of kidney aging. These findings were subsequently confirmed by Cohen et al., who showed that the severity of glomerulosclerosis was reduced in aged p16-INK-ATTAC, which was associated with better preservation of podocytes in 28-month-old mice [93]. Another senescence-depletion model is the p16-3MR mouse, which was originally developed by Demaria et al. [105]. In this system, the p16 promoter drives the expression of a reporter (Renilla luciferase) together with the herpes simplex virus thymidine kinase, which induces apoptosis when cells are exposed to ganciclovir. Aging experiments in p16-3MR mice showed that ganciclovir induced clearance of senescent cells from the kidney, which was associated with improved renal function in old mice [106].

Taken together, these findings using selective killing of p16-expressing cells indicate a functional role of senescent cells in chronological kidney aging. As the p16 promotor is not exclusively activated in senescent cells though, but also in non-senescent cells (e.g., macrophages and other cell types), it is currently not possible to attribute all observed effects to the elimination of senescent cells.

### Pharmacological approaches to senolysis

Johmura et al. established a senolytic strategy where targeted inhibition of kidney-type glutaminase very efficiently eliminated senescent cells in aged mice [107]. This was associated with improved glomerulosclerosis, less inflammation, and better kidney function. Baar et al. took another approach using a strategy of interference with the molecular interaction between p53 and the transcription factor FOXO4 to trigger a release of p53 from the nucleus with subsequent induction of cell-intrinsic apoptosis. According to this strategy, elimination of senescent cells is achieved by a modified FOXO4/p53-interfering peptide (FOXO4-DRI). Interestingly, FOXO4-DRI administration significantly reduced the load of senescent cells in kidneys from progeroid or naturally aged mice, which was paralleled by improved kidney function and less interleukin-6 expression, a typical SASP marker [106].

Similar to the administration of the peptide FOXO4-DRI, elimination of senescent cells can be achieved by the administration of small molecule compounds, which also interfere with the cell’s over-activated survival and anti-apoptosis mechanisms [108]. Such ‘senolytic’ compounds, often repurposed from the cancer field, have been used in a variety of experimental studies. While most of the studies tested senolytics in stress and injury models of age-related disease, there are only limited data on the effects during natural kidney aging. For example, Yousefzadeh et al. reported beneficial effects of the flavonoid fisetin, reducing renal cellular senescence, expression of SASP genes, and preserving renal histology [109]. Importantly, besides its senolytic properties fisetin has additional effects, such as inhibition of the NLRP3 inflammasome and mitochondrial protection, which could contribute to the observed effects [110–112]. Mitochondrial integrity and intact mitophagy are crucial for healthy kidney aging. Antiaging strategies addressing abnormal mitochondrial function and decreased mitophagy have the potential to delay cellular senescence and improve kidney aging [113].

ABT-263 is another senolytic compound, which overcomes anti-apoptotic survival by inhibiting B-cell lymphoma (Bcl) 2/w/xL. Mylonas et al. found a significant reduction in senescence markers in kidneys of aged mice after treatment with ABT-263. When old mice were additionally challenged with ischemia/reperfusion as a model of AKI, kidneys showed better repair and reduced fibrosis [114]. Additional evidence supporting the potential of ABT-263, has been provided in a cisplatin model of CKD, in which the senolytic effects of ABT-263 attenuated fibrosis and improved tubular repair [110]. In another report, treatment of old mice with ABT-263 caused a significant reduction of renal SA-β-GAL but also a concomitant increase in p16 expression, possibly indicating a secondary cellular stress response [115].

An additional flavonoid, quercetin, in combination with the kinase inhibitor dasatinib (DQ), is a frequently used senolytic approach, which has even already entered the clinical trial arena [116,117] In a small proof of principle trial with diabetic patients treatment with DQ decreased skin p16 and p21 expressing cells and reduced circulating SASP factors [116]. In non-human primates, long-term DQ treatment reduced several markers of aging, accompanied by lower blood urea nitrogen as a potential sign of improved renal function [118]. In mice, the administration of DQ up-regulated renal expression of anti-aging protein α-Klotho, which was also mirrored in patients treated with DQ [119].

### α-Klotho

α-Klotho is expressed in the distal tubule of the healthy kidney, where it functions as a co-receptor for fibroblast growth factor 23 and is also cleaved and released to the circulation, exerting many different effects in a variety of organs [120]. In the kidney, α-Klotho is crucial for essential renal functions such as phosphate homeostasis and mineral metabolism but also for cellular protection against oxidative damage, cytotoxic injury, and fibrosis. The renal expression of α-Klotho decreases with age, and hypomorphic α-Klotho mice show accelerated aging and an increased load of cellular senescence in the kidney [121]. Based on this antagonistic interrelationship between α-Klotho and cellular senescence, it is noteworthy that concomitant genetic ablation of p16 rescues the accelerated aging phenotype in hypomorphic α-Klotho mice [122]. As 16 deletion did not rescue mice with a complete α-Klotho knockout, it was concluded that p16 represses the physiological α-Klotho expression.

### Infectious diseases

Viral infections, which often induce DNA damage, can stimulate premature cellular senescence in a variety of cell types. It has been shown that severe forms of SARS-CoV-2 infections were associated with the induction of cellular senescence not only in infected pulmonary cells but also in brain tissue and immune cells [123–125]. Although there are no published data on the specific impact of SARS-CoV-2 on cellular senescence in the kidney, it can be speculated that virus-evoked cellular stress acts as a pro-senescent trigger on infected renal cells [126]. The potential importance of cellular senescence in this context has been highlighted by preclinical data, in which targeted elimination of senescent cells improved functional outcome in COVID-19 models [127]. More detailed kidney studies will explore the interplay between viral infection and cellular senescence to evaluate the potential of anti-infective strategies in delaying renal aging.

## Tubulointerstitial fibrosis and cellular senescence

Tubulointerstitial fibrosis (TIF) is part of the senile nephrosclerosis pattern seen in aging kidneys, but it is also the shared endpoint of most forms of CKD [128]. TIF is characterized by the excess accumulation of extracellular matrix in the renal interstitium—the space between the tubules in the kidney—and is closely associated with functional deterioration [129].

Numerous studies have demonstrated a correlation between pathways of renal senescence and TIF in murine and rat models. For instance, Braun et al. demonstrated that deleting the p16 coding gene significantly reduced TIF after ischemia/reperfusion injury, thereby improving renal function and kidney transplant survival [130]. Similar findings were independently reported by other groups not only for the kidney but also for cardiac ischemia/reperfusion [131,132]. Deletion of p16 also improved TIF in a stress-induced premature senescence model caused by deficiency of Bmi-1, an oncogene involved in chromatin remodeling and stem cell renewal [133]. Further evidence for a connection between TIF and cellular senescence was provided by several studies showing that experimental senolysis resulted in a reduction of TIF. For example, ABT-263 reduced TIF after ischemia/reperfusion in irradiated mice and chronologically aged mice, as well as in mice challenged with repeated low dose of cisplatin [110,114]. A functional relationship between cellular senescence and TIF was also suggested by a mouse model with conditional deletion of the scaffold protein salvador homolog 1 (Sav1) in proximal renal tubules. Deletion of Sav1 lead to up-regulation of key senescence markers, which was associated with Stat3 activation, and a strong increase of pro-fibrotic effector pathways, implicating a correlated pathogenesis [134].

Senolysis studies sometimes show heterogeneous effects on TIF depending on the senolytic strategy and the model. This is illustrated by a report using a parallel analysis of p16-3MR mice and application of FOXO4-DRI in a model of folic acid-induced AKI. Both strategies were able to efficiently eliminate senescent cells in the kidney. However, while TIF was reduced in the p16-3MR mice, FOXO4-DRI treatment did not counteract TIF development, suggesting a specific cell-type dependency and challenging the direct interrelationship of cellular senescence and TIF [98].

### TGF-β1, a renal SASP candidate

Recently, O’Sullivan et al. performed an elegant study, in which depletion of senescent cells with ABT-263 reduced TIF in a ureteral obstruction model. The authors introduced protein disulfide isomerase family A member 3 (PDIA3) dependent signaling as a pathway connecting epithelial cell senescence and transforming growth factor β1 (TGF-β1)-mediated fibroblast activation [77]. Although not all senescent renal cells secrete TGF-β1, it is often regarded as a typical SASP component of kidney aging. TGF-β1 is decisive in many forms of CKD as it is a potent renal fibroblast activator and pro-fibrotic mitogen [135,136]. In the context of senescence, TGF-β1 might act as a reciprocal activator, which is secreted by senescent cells and induces further senescence in surrounding cells. TGF-β1 secreted locally by podocytes may lead to pro-senescent changes in glomerular endothelial cells via transcriptional activation of p21 and nuclear translocation of p16, both in a Smad3 dependent manner [137]. Because cell senescence and secretion of TGF-β1 is not always co-activated, variable regulators must be involved. Wnt9a was reported as an interconnecting pathway which exacerbates the induction of tubular cell senescence, induces the production of TGF-β1, and promotes TIF [138]. A novel interesting candidate along these lines is Legumain, a protein down-regulated with aging and typically contributing to lysosomal function in renal proximal tubular cells. Tubular deletion of Legumain induced premature senescence, a strong up-regulation of TGF-β1 and accelerated TIF. Importantly, these changes were partially rescued by parallel knockdown of p16 [139].

## The role of cellular senescence in the diseased kidney

Despite the growing interest in cellular senescence as a driver of CKD, data linking specific kidney diseases to cellular senescence remains limited in humans. Subsequently, we will summarize the current literature covering human studies according to major kidney disease groups and substantiate these studies with preclinical results from animal experiments.

### Diabetic nephropathy (DN)

Diabetes mellitus, including Type 1 and Type 2, is considered the leading cause of CKD worldwide. The pathogenesis of diabetic nephropathy (DN) is complex and multifaceted. Specifically, approximately a third of people with Type 1 diabetes and up to half of all patients with Type 2 diabetes experience DN throughout their life. At the histological level, DN is characterized by distinct changes such as thickening of the tubular and glomerular basement membranes, fusion and loss of podocyte foot processes, and expansion of the mesangial matrix [140,141]. Kidney aging and DN share several pathophysiological mechanisms, including the accumulation of advanced glycation end products (AGEs), which trigger oxidative stress, inflammation and pro-senescent conditions [142,143]. Increased markers of senescence were reported by several groups in biopsies or nephrectomy specimen together with signs of DN [144,145]. SA-β-GAL and p16 in renal tubules directly correlated with blood glucose levels, while glomerular p16 expression (ranging from 1 to 3%) was associated with proteinuria, a hallmark of DN [145]. A longitudinal study with Type 2 diabetes patients showed an association between the progression of DN and increased DNA damage, as evidenced by the comet assay and γH2AX-positivity in leukocytes, increased levels of p21 in the urine, and increased SASP markers (IL-6 and TGF-β1) [146].

Experimentally, a clear correlation of senescent tubular cells with CKD progression was observed in streptozotocin-treated diabetic animals [147,148]. While the underlying connection is not completely clear, metabolic alterations seem likely. Counteracting high glucose reabsorption of proximal tubular cells by a sodium-glucose cotransporter-2 inhibitor (SGLT2i), markedly reduced the development of cellular senescence [149]. It was suggested that this effect could at least be partially mediated by enhanced SGLT2i-dependent production of β-hydroxybutyrate. This concept was supported by data showing that podocyte senescence and diabetic glomerulopathy were successfully reduced by β-hydroxybutyrate administration [150]. A geroprotective potential for repurposing of SGLT2is, which have shown significant therapeutic benefits in clinical DN and CKD, has thus been suggested [151].

Unexpected findings indicated an active role of the complement system in DN acting through altered DNA methylation on tubular cells [152,153]. The HDAC inhibitor, valproic acid, attenuated diabetes-induced up-regulation of complement C5a receptors, blocked the development of tubular cellular senescence, and diminished functional damage of DN [153]. Similar results were obtained by blockade or knockout of the C5a receptor C5aR1, reducing senescence markers and SASP expression as well as restoring α-Klotho expression in murine DN [153]. An additional link between epigenetic changes, cellular senescence, and progression of DN is provided by the observation that hyperglycemia modulates the promoter region of CDKN1A (p21 gene), leading to sustained p21 expression and subsequent cellular senescence as a potential driver of DN [154]. Although there are still many open questions, these findings support a role for cellular senescence in the development and progression of DN (reviewed in [155]).

### Arterial hypertension

Hypertension, the second most dominant entity in the development of CKD, has also been linked to cellular senescence. Westhoff et al. not only found increased renal p16 expression in patients with hypertensive nephrosclerosis but also established a causal relationship by using antihypertensive medication to attenuate senescence induction in rats [156]. In a commonly used rat hypertension model, stroke-prone spontaneously hypertensive rats (SHRSPs), kidneys of male rats were characterized by an accelerated accumulation of senescence markers, which was paralleled by enhanced TIF and tubular damage [157]. Associative evidence was provided by a clinical study measuring p16 in urinary extracellular vesicles (EVs) of hypertensive (essential and renovascular hypertension) patients and healthy volunteers. EVs that were derived from different tubular segments showed a clear association between p16 and clinical hypertension [158].

To elucidate the functional role of cellular senescence in hypertension, senolysis was used in INK-ATTAC mice after chronic exposure to low-dose angiotensin II, which induced several markers of senescence and SASP. Importantly, INK-ATTAC-driven elimination of p16-high cells did not alter blood pressure but prevented SASP activation and inflammatory changes [159]. These results suggest that senolytic strategies could be considered for preventing hypertension-induced kidney damage. Interestingly, some commonly used antihypertensive medications, for example, hydrochlorothiazide, hydralazine, and inhibitors of the renin–angiotensin–aldosterone system, seem to slow down the development of kidney cellular senescence [156,160]. Along these lines, recent evidence points towards the possibility, that the antihypertensive medication rilmenidine, an I1 imidazoline receptor activator, might have broad anti-aging effects in the kidney, possibly mediated via transcriptional changes similar to caloric restriction [82].

### Glomerulonephritis

Auto-inflammatory kidney diseases comprise the third large group of CKD entities, in which immune-mediated processes disrupt glomerular structures, leading to glomerulonephritis with proteinuria, hematuria, and loss of renal function. Biopsy studies in patients with Immunoglobulin A nephropathy (IgAN), the most common glomerulonephritis, demonstrated a correlation between disease severity and increased senescence in renal epithelial cells [161]. When ESRD patients with IgAN were followed after receiving a kidney transplant, an association was found between accelerated senescence (increased p16) and the development of TIF. Interestingly, the p16 load correlated with a polymorphism in the complement factor H (CFH) gene, a regulator of the complement system, which might again point towards an interdependent senescence-complement mechanism [162].

Lupus nephritis (LN) is a common complication in systemic lupus erythematosus (SLE), leading to 10% of ESRD cases. For patients with active LN, the presence of p16-positive cells was associated not only with lower eGFR but also with increased renal fibrosis and CD8^+^ T cell infiltration [163]. In the lupus-prone MRL/lpr mouse, severity of LN strongly correlated with glomerular SA-β-GAL positivity, which was reversible by dexamethasone treatment [164]. In the same mouse model, treatment with the senolytic fisetin reduced the number of senescent tubular cells, attenuated SASP expression, and reduced TIF [165].

Interestingly, the B-cell activating factor (BAFF), which supports autoreactive B cells in the pathogenesis of LN, has recently been highlighted as a novel SASP factor and a driver of pro-senescent changes [166]. Given these findings, antagonizing senescence-dependent disease progression might offer therapeutic perspectives for Belimumab, a biologic BAFF inhibitor, already licensed for the treatment of SLE and LN.

Taken together, despite many unresolved aspects, the existing data indicates that cellular senescence is both, a marker and driver of CKD.

## Conclusion and future directions

**Figure 4:**
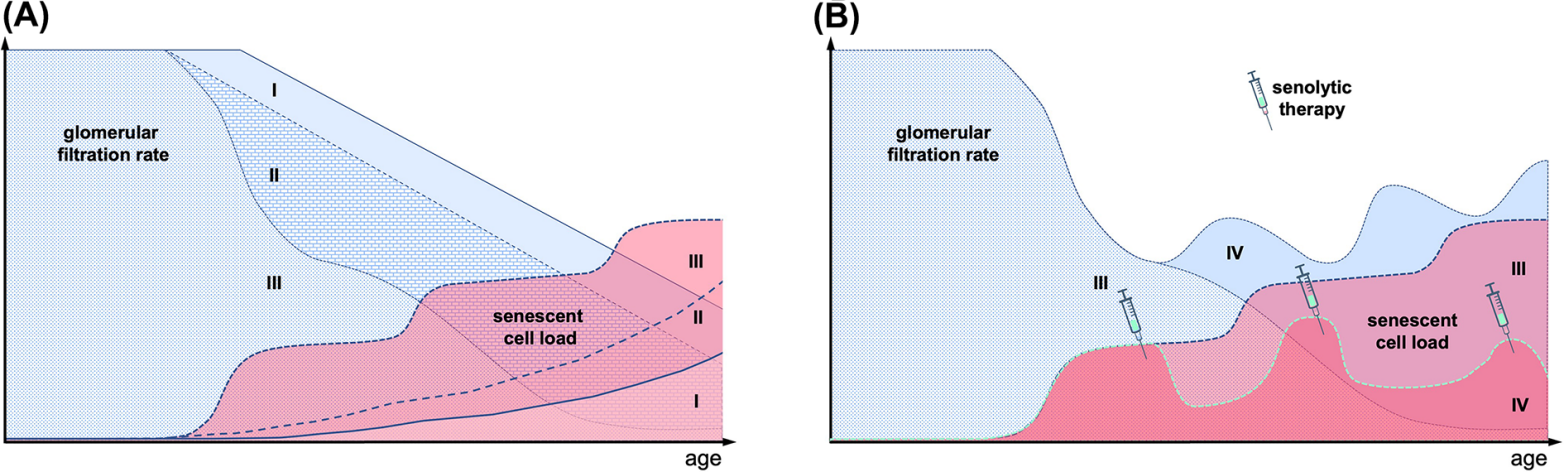
Hypothetical trajectories of renal function and senescent cell load (**A**) Loss in glomerular filtration rate with natural aging (I) and accelerated aging with increased senescent cell load (II, III). (**B**) Repetitive senolytic drug treatment diminishes the senescent cell load, leading to improved glomerular filtration rate.

The aging kidney is characterized by changes in function and structure. Some of these changes overlap with pathological alterations seen in CKD. There are correlative clinical data and growing experimental evidence that cellular senescence is not only a marker of aging and CKD but an active contributor. Cellular senescence may act as a driver of failed repair, accelerated nephrosclerosis, TIF, CKD progression, and loss of renal function (Figure 4A). Antagonizing cellular senescence, for example, by senolysis, is expected to alleviate these changes (Figure 4B). As the contribution of cellular senescence to natural aging and age-related disease is context-specific, the effect size of any anti-senescence strategy will be heterogeneous. It will be important to explore, how different inducers and effectors of renal cellular senescence, like oxidative stress, telomere shortening, DNA damage, α-Klotho expression, complement activation, metabolic changes, and TGF-β1 signaling, act in concert, to take advantage of a plethora of potential therapeutic options. Besides senolysis, these options also include repurposing existing drugs, for example, certain antihypertensives, antidiabetics (e.g., Metformin, SGLT2i), mTOR inhibitors and broader anti-inflammatory substances. Modulating cellular senescence might also be possible by non-pharmacological strategies, which modify the sum of environmental exposures across our life [167]. This may include approaches already used in general practice, such as changes in nutrition (e.g., low salt diet) [168], anti-infective strategies (e.g., vaccination against COVID-19) [169] and where possible avoidance of environmental pollutants (e.g., cadmium) [170], factors which all have been proposed to protect the kidney from developing cellular senescence.

## Data Availability

N/A

## Abbreviations

AKI: acute kidney injury
BAFF: B-cell activating factor
cGAS-STING: cyclic GMP-AMP synthase-stimulator of interferon genes
CFH: complement factor H
CKD: chronic kidney disease
DDR: DNA damage response
IgAN: immunoglobulin A nephropathy
LN: lupus nephritis
PDIA3: protein disulfide isomerase family A member 3
SASP: senescence-associated secretory phenotype
SGLT2i: sodium-glucose cotransporter-2 inhibitor
SHRSP: stroke-prone spontaneously hypertensive rat
SLE: systemic lupus erythematosus
TGF-β1: transforming growth factor β1
TIF: tubulointerstitial fibrosis
